# Two temperature-dependent membrane fluidity regimes in gram-positive bacteria

**DOI:** 10.1128/msphere.00095-26

**Published:** 2026-06-11

**Authors:** Aurélien Barbotin, Dimitri Juillot, Paprapach Wongdontree, Rut Carballido-López

**Affiliations:** 1 Université Paris-Saclay, INRAE, AgroParisTech, Micalis Institute, Jouy-en-Josas, France; The University of Iowa, Iowa City, Iowa, USA

**Keywords:** plasma membrane fluidity, thermoadaptation, TIR-FCS, gram-positive bacteria

## Abstract

Temperature changes directly affect the mobility of molecules within a biological membrane, increasing or decreasing its fluidity. Plasma membrane fluidity is a fundamental physical property that governs bacterial cell physiology, influencing processes such as protein function, respiration, and antibiotic uptake. It is widely accepted that bacteria actively maintain a constant membrane fluidity in response to temperature fluctuations to preserve membrane function. This process, known as membrane fluidity homeostasis, occurs through regulated remodeling of lipid composition. Here, we used an assay based on total internal reflection-fluorescence correlation spectroscopy to directly quantify membrane fluidity in the gram-positive model bacterium *Bacillus subtilis* and two gram-positive pathogens, *Streptococcus pneumoniae* and *Staphylococcus aureus*, across a temperature range of 20°C to 37°C. Instead of the expected constant membrane fluidity, we revealed a two-component regime: membrane fluidity is maintained at low temperatures (<26°C) but freely increases at higher temperatures. Far from contradicting previous evidence about thermoadaptation in bacteria, and especially in *B. subtilis*, our results demonstrate that their usual interpretation is incorrect and provide a framework for studying membrane fluidity across bacterial species.

**IMPORTANCE:**

Temperature changes affect the physical properties of the plasma membrane. Typically, a reduction in temperature causes a less fluid and thus more viscous, membrane. It has been long established that bacteria such as the model bacterium *Bacillus subtilis* adapt their membrane composition to maintain membrane fluidity constant under changing temperatures, to survive environmental changes. By directly quantifying membrane fluidity across temperatures in *B. subtilis* and two other gram-positive bacteria, we found that this long-standing view is not true: fluidity is maintained only at low temperatures (<26°C), while at higher temperatures, it increases linearly with temperature. Our findings present a conceptual advance that broadens our understanding of thermoadaptation and refines the current model of membrane fluidity homeostasis in bacteria.

## OBSERVATION

Membrane homeostasis is a process that involves remodeling the plasma membrane in response to external stimuli, such as changes in temperature. It has been observed in numerous living organisms (1), and one common explanation is the need to keep membrane fluidity constant. In bacteria, membrane fluidity changes have been linked to a wide variety of processes like responses to physical or chemical stresses, protein folding, respiration, and antibiotic uptake and resistance (2, 3). One of the best-characterized aspects of bacterial membrane fluidity is its homeostasis in response to temperature fluctuations. In gram-negative bacteria such as *Escherichia coli*, membrane fluidity homeostasis was measured as a gradual increase in membrane fatty acid (FA) unsaturation as temperature decreased (4). The mechanism of adaptation to low temperatures in gram-positive bacteria such as *Bacillus subtilis* (5) is more complex: cold shock is sensed by the DesK/DesR two-component system, which activates transcription of the gene encoding the FA desaturase Des (6), and long-term membrane adaptation further involves FA branching and chain shortening (5). Membrane FA analysis has long remained the gold standard to evaluate membrane fluidity homeostasis, despite growing evidence of the role of other factors like lipid headgroups (7) or proteins (8) on the physical properties of the membrane. Another standard approach, which consists of using environment-sensitive probes such as diphenylhexatriene or Laurdan (9), produced conflicting results (10). This is not entirely surprising, as environment-sensitive probes report on complex signals (11) that correlate with membrane fluidity only under specific assumptions that are often not verified in biological experiments (12).

Recent direct, quantitative measurements revealed that plasma membrane fluidity in *B. subtilis* is approximately twofold lower at low temperatures (20°C–23°C) than at 37°C (13, 14). This observation contradicted the substantial body of literature that uncovered thermoadaptation mechanisms in this bacterium. Here, we sought to resolve this apparent contradiction and applied a recently developed technique based on total internal reflection-fluorescence correlation spectroscopy (TIR-FCS) (14) to directly quantify membrane fluidity across a range of temperatures in *B. subtilis* and two other gram-positive bacteria. We found two distinct operating regimes: at low temperatures (<26°C), membrane fluidity is maintained constant by the established homeostatic feedback mechanisms; in contrast, at higher temperatures, membrane fluidity increases linearly with temperature.

**Fig 1:**
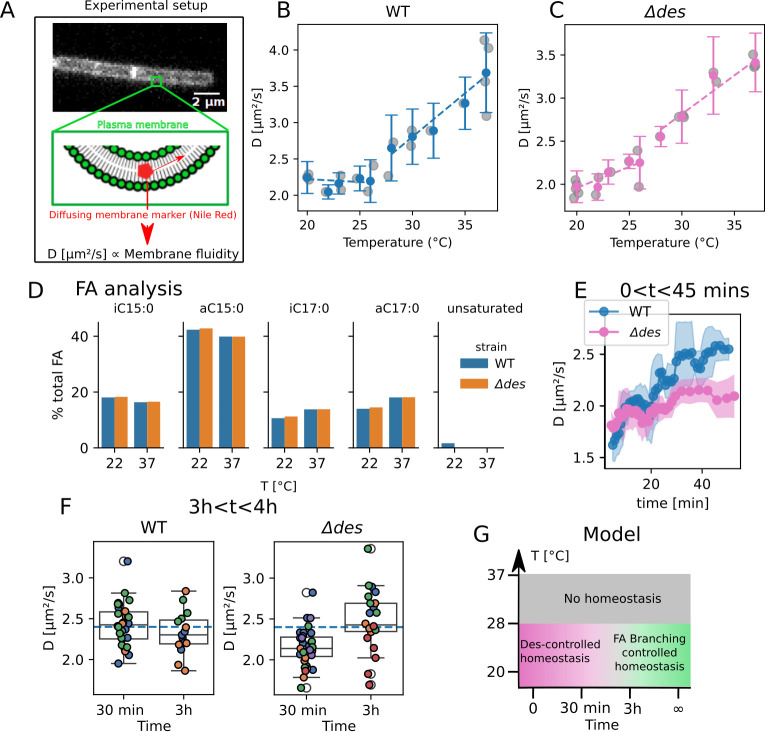
Temperature-dependent membrane fluidity homeostasis in *B. subtilis*. (**A**) Schematic representation of the methodology. Membrane fluidity is measured as the diffusion coefficient (*D*) of the membrane marker Nile Red in the plasma membrane. (**B and C**) Membrane fluidity in wild-type (WT) (**B**) and Δ*des* (**C**) *B. subtilis* cells measured 25–70 min after transfer at the target temperature. Blue: mean *D* ± SD; gray dots: median D of individual replicates. (**D**) Relative FA composition of WT and Δ*des* strains at 37°C and 45 min after cold shock at 22°C. FA types represented are iso (iC) or anteiso (aC) with saturated carbon chains of length 15 or 17 and unsaturated FA (mean of *n* = 2). (**E**) Time-dependent membrane fluidity recovery in WT (blue) and Δ*des* (pink) cells after a shift from 37°C to 20°C. (**F**) Steady-state membrane fluidity in WT (left) and Δ*des* (right) *~*30 min and ~3 h after cold shock. (**G**) Proposed model of temperature-dependent membrane fluidity control in *B. subtilis*.

We quantified membrane fluidity as the diffusion speed of the membrane marker Nile Red in live wild-type *B. subtilis* cells using TIR-FCS (14) (Fig. 1A). Cells growing at 37°C were shifted to a target temperature between 20°C and 37°C. We first measured membrane fluidity shortly after transfer (25–70 min, after complete cold shock recovery (14) . Surprisingly, membrane fluidity in *B. subtilis* was neither constant nor proportional to temperature. Instead, we observed two operating regimes: membrane fluidity was constant below 26°C and evolved linearly with temperature above this temperature (Fig. 1B). To confirm that the well-studied thermosensing pathway of *B. subtilis* contributes to this behavior, we examined a mutant lacking *des*, which encodes the FA desaturase Des, the fast responder that immediately desaturates membrane FA after a cold shock (5). We quantified membrane fluidity in a Δ*des* mutant across the same temperature range tested in wild-type cells. Fluidity was no longer constant below 26°C (Fig. 1C), confirming the role of Des in membrane fluidity homeostasis at low temperatures. Analysis of FA composition revealed that the only notable difference between wild-type and Δ*des* cells growing exponentially either at 37°C or 45 min after a cold shock at 22°C was the appearance of unsaturated FA exclusively in wild-type cells after cold exposure (Fig. 1D), in agreement with the fluidity differences observed between the two strains at low temperature. Both strains exhibited a similar reduction in overall FA carbon chain length after cold shock (Fig. 1D), as previously reported (15), consistent with early stages of the long-term membrane fluidity adaptation.

Measuring the dynamics of cold shock (37°C → 20°C) recovery in both wild-type and Δ*des* cells, we observed, as expected, that membrane fluidity recovered only partially and much more slowly in the Δ*des* mutant than in the wild type (Fig. 1E). We concluded that the Δ*de*s mutant fails to maintain membrane homeostasis at low temperatures because of its inability to rapidly increase membrane fluidity. We next verified if this defect is transient, as predicted by the literature indicating that long-term membrane homeostasis relies on FA branching and chain shortening and not on unsaturation. To test this, we quantified membrane fluidity in wild-type and Δ*de*s cells immediately after cold shock and again after ~3 h (about one doubling time) at 20°C. While wild-type cells exhibited the same membrane fluidity regardless of the time spent at 20°C, Δ*de*s cells recovered the wild-type fluidity after 3 h at 20°C (Fig. 1F). Membrane fluidity of wild-type cells remained comparatively lower at 20°C than at 37°C even for cells grown at 20°C for 24 h (data not shown). Based on these observations, we propose a new model of membrane fluidity homeostasis that completes the current model and reconciles conflicting observations (Fig. 1G). Our findings are consistent with the notion that the two known mechanisms of fluidity homeostasis, FA unsaturation and branching/chain shortening, are indeed activated at different timescales following a cold shock to maintain membrane fluidity. However, our data revealed that above a critical temperature, which we estimate around 26°C (Fig. 1B), membrane fluidity is not maintained constant.

**Fig 2:**
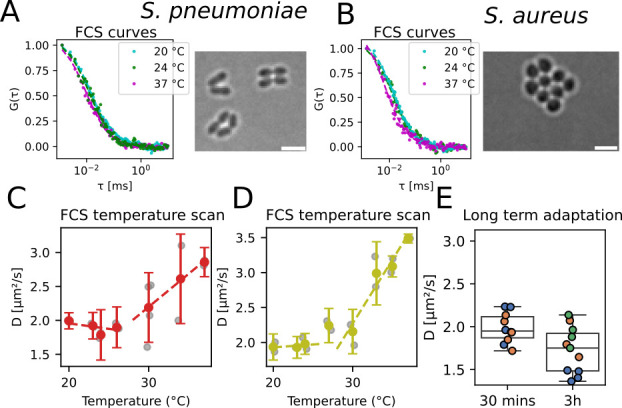
FCS temperature scans in *S. pneumoniae* (**A and C**) and *S. aureus* (**B, D, and E**). (**A and B**) Left: representative FCS curves at different temperatures. Right: bright-field images (scale bar, 2 µm). (**C and D**) Temperature-dependent membrane fluidity. (**E**) Steady-state membrane fluidity *~*30 min after cold shock or ~3 h after cold shock in *S. aureus*.

To assess whether this model is specific to *B. subtilis* or extends to other gram-positive bacteria, we performed analogous TIR-FCS temperature scans in two gram-positive mesophilic pathogens, *Staphylococcus aureus* and *Streptococcus pneumoniae*. Both bacterial species displayed a membrane fluidity profile similar to that of *B. subtilis*: membrane fluidity remains constant below a critical temperature and increases linearly at temperatures above this threshold (Fig. 2A through D). We verified that steady-state membrane fluidity was reached after 30 min in *S. aureus* (Fig. 2E), as observed in *B. subtilis*, ensuring that the two-component effect in *S. aureus* was not due to a slower recovery caused by lack of desaturase Des.

In all three bacterial species, the temperature threshold between the two regimes seems to be around 26°C, and the diffusion coefficients are remarkably similar (ranging from 2 to 2.5 µm²/s at lower temperatures to ~3.5 µm²/s at higher temperatures). This could hint at a conserved membrane fluidity control mechanism across all three species, consistent with recent reports that identified thermosensor proteins similar to those of *B. subtilis* in *S. aureus* and *Bacillus anthracis* (16).

Through quantitative measurements of membrane fluidity, we uncovered the existence of two distinct operating regimes of membrane fluidity in gram-positive bacteria, instead of one as established in the field. This discovery does not contradict earlier observations, in particular cold-induced changes in membrane composition, but rather refines and extends the current model. The temperature threshold between the two regimes, which we estimate to be around 26°C in *B. subtilis*, is in line with the previously reported activation temperature of the thermosensor DesK (17).

Our finding raises many intriguing questions. Are these two regimes also present in gram-negative bacteria, contrary to what was previously thought (18)? We observed the same regimes in two gram-positive bacteria besides *B. subtilis*; how do they maintain membrane fluidity at low temperatures?

Most interestingly, what is the point of these two operating regimes? In *B. subtilis*, the thermosensor DesK is thought to measure membrane thickness and not fluidity directly (5). Perhaps membrane thickness exhibits the same two-regime behavior, and a similar pattern may apply to many other physical parameters of the membrane. Control of membrane fluidity may simply be a byproduct of the control of another, more important biophysical parameter like membrane compressibility (19). According to the homeophasic adaptation hypothesis, membrane fluidity homeostasis at low temperatures could be simply necessary to prevent phase separation (20) and its detrimental effects to cell physiology (21). The constant fluidity at low temperatures would then simply correspond to a minimum fluidity that prevents phase separation.

Diffusion speed of membrane marker Nile Red was measured with TIR-FCS (12). Each acquisition of 50,000 frames was about 1 min long. Raw diffusion coefficients were divided by a factor specific to the shape of each bacterium (*B. subtilis*: 1.54; *S. aureus*: 2.38; and *S. pneumoniae*: 2.19) estimated from simulations. Detailed methods are provided in the Supporting information.

## Data Availability

Research data and code to generate the figures are available on Zenodo at https://zenodo.org/records/17820983.

## SUPPLEMENTAL MATERIAL

Supporting information msphere.00095-26-s0001.docx Extended methods.

ASM does not own the copyrights to Supplemental Material that may be linked to, or accessed through, an article. The authors have granted ASM a non-exclusive, world-wide license to publish the Supplemental Material files. Please contact the corresponding author directly for reuse.
